# An Analysis of Pediatric Scar Progression Over Time

**Published:** 2018-04-27

**Authors:** Blaire Slavin, Roberta Torres, Anne C. Fischer

**Affiliations:** ^a^University of Miami, Miami, Fla; ^b^Florida Atlantic University, Boca Raton

**Keywords:** infantile scar growth, multigenerational, pyloric stenosis, pyloromyotomy, right upper quadrant

## Abstract

**Objective:** The advances in surgical approaches for a pyloromyotomy have all focused on creating smaller incisions from a right upper quadrant now to a laparoscopic umbilical incision. A key assumption is that the final scar retains the size of the original incision as the child matures. Our case reports on a family with several members, now adults, with the same surgery and same surgeon who had the right upper quadrant incision as infants to elucidate the extent of how infantile scars grow over time, significantly exceeding the original incision. **Methods:** We evaluated the various pyloromyotomy scars of our newborn patient, his maternal grandmother, and his two maternal twin aunts. One aunt (#1) was of normal stature, whereas her twin (#2) never went through a full vertical growth phase due to being stunted by Cornelia de Lange syndrome. For each member, we compared the length of the original incision with the current scar length to determine how much the scar has grown over time. **Results:** Significant scar growth was seen in the grandmother and aunt 1. In contrast aunt 2’s scar did not grow significantly due to her stunted vertical growth from Cornelia de Lange syndrome. **Conclusions:** This case supports the notion that surgical incisions in infants grow more substantially than realized with age, resulting in larger scars than anticipated. Our findings suggest the reason why the laparoscopic pyloromyotomy has been popularized due to its incisions being so small that they continue to present a cosmetic advantage over time.

Infantile hypertrophic pyloric stenosis (IHPS) is a common surgical condition in newborns typically between 2 and 8 weeks of age.^1^ The narrow pyloric channel results in gastric outlet obstruction.^2^

Several surgical approaches to pyloromyotomy have evolved over the years. The first pyloromyotomy was performed by Ramstedt in 1912 and typically reported a 3 cm transverse right upper quadrant (RUQ) incision, which was the most popular incision until the late 1980s.^2^ Although the open RUQ pyloromyotomy left a small scar as a baby, the scar was not appreciated as increasing in size as the child reached adulthood. In 1986, Tan and Bianchi proposed an equally successful approach to an open pyloromyotomy via a supraumbilical incision that would leave a smaller, less noticeable scar.^3^ This supraumbilical incision uses the proximity to the umbilical folds to trick the eye into appreciating the scar to be a skinfold. The 2000s saw the popularization of the laparoscopic pyloromyotomy, which requires two 3 mm left/right paramedian stab incisions and 1 imperceptible 5 mm or less umbilical incision hidden in the umbilical skinfolds.^1^ Because cosmesis is so important, the laparoscopic approach currently supersedes open pyloromyotomy as the most popular surgical approach to treat IHPS.^4^ However, many surgeons still advocate the open procedures since they claim the scars are small, overlooking their potential for growth.

Our case report utilizes a family with several members treated by an RUQ pyloromyotomy as infants to show how much infantile scars can actually grow. Although the RUQ incision is rarely seen now due to the evolution of the surgical approach to pyloromyotomy, the comparison of this simple, transverse incision from infancy to adulthood effectively illustrates how much a scar can grow. The change in surgical approach over time and the extent of scar growth into adulthood is documented in this multigenerational family who has a history of IHPS in 3 generations.

## CASE REPORT

Our patient was a male infant born at 36 weeks who by 4 weeks of age displayed a 5-day history of nonbilious, nonbloody emesis either directly after his feeds or several hours later. A laparoscopic pyloromyotomy was attempted, which was converted into a supraumbilical open pyloromyotomy due to inability to tolerate sufficient insufflation. The size of the surgical incision for the laparoscopic approach was 8 mm to accommodate a 5 mm umbilical port, followed by a 3 cm incision for the supraumbilical conversion.

The patient's family history was significant for pyloric stenosis across 3 generations since the newborn, his maternal grandmother, and his 2 maternal twin aunts all had IHPS. The grandmother had an oblique RUQ pyloromyotomy at 4 weeks of age in 1959 with an original scar incision of ∼4 cm. Since her procedure 58 years ago, the grandmother's scar has grown 8 cm in length, with a current scar length of 12 cm. Aunt 1 underwent a transverse RUQ pyloromyotomy at 4 weeks of age in 1994, with an original incision length of 4 cm and a current scar length of 8 cm. Aunt 2 required a transverse RUQ pyloromyotomy at 6 weeks of age with an incision of 4 cm and a current scar length of 4 cm. Both aunts had the same surgeon and the same diagnosis within 2 weeks of each other. Aunt 2’s scar size of 4 cm was smaller than her twin sister's 8 cm scar due to her short stature from Cornelia de Lange syndrome (Table 1).

## RESULTS

**Figure 1:**
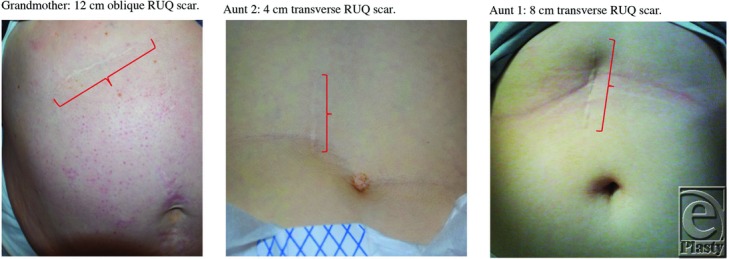
Significant growth in scar length since initial procedure, as seen in grandmother and aunt 1 but not aunt 2, due to stunted growth from Cornelia de Lange syndrome. RUQ indicates right upper quadrant.

**Figure 2:**
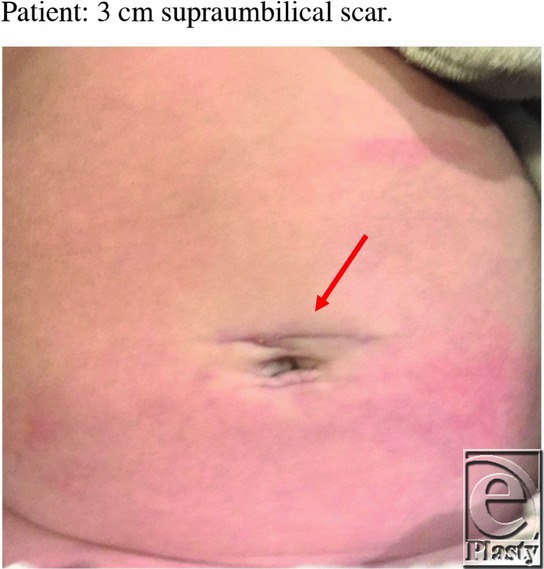
Twelve-week-old patient's scar from supraumbilical incision. No scar growth detected because he is still a newborn.

See Table 1 and Figures 1 and 2.

## DISCUSSION

The case that we present serves as a unique opportunity to analyze scar sizes following a procedure to correct the same disease, given that the patients considered are all from the same family with the same diagnosis and similar surgery. The body of scientific literature discussing scar growth from procedures performed during infancy is relatively limited. In 1860, James Paget challenged the assertion that childhood scars do not grow with age. In a lecture to the Royal College of Surgeons of England, he stated, “The scar of the child, when once completely formed, commonly grows as the body does, at the same rate, and according to the same general rule.”^5^ In 1873, Adams supported Paget's words when he observed that a 21-month-old patient who underwent a surgical procedure to correct congenital varus of both feet had scars that increased in length by approximately 2.5 cm in the span of 7 years.^6^ In terms of scarring resulting specifically from open RUQ pyloromyotomy, Harmon^4^ noted that the adult patient's scar had grown in both length and width, puckered because of adherence of skin to abdominal muscular fascia, and deepened because of an increase in thickness of subcutaneous adipose tissue since infancy. Harmon's^4^ descriptions were also observed in our case report, as seen in the patient's grandmother whose oblique RUQ scar grew 8 cm in length since the procedure was performed in infancy. This was similar to the 4 cm scar growth seen in aunt 1. The grandmother and aunt 1 are perfect illustrations of the potential scar growth often underestimated in surgery on infants. Because of her stunted growth, aunt 2’s scar did not see the same drastic growth as her twin, aunt 1. Aunt 2’s body did not grow significantly, nor did her scar.

The drive to modify the surgical technique to pyloromyotomy over the years is largely due to wanting to minimize the size of the initial scar, yet the potential for scar growth into adulthood is still highly unappreciated. Haricharan et al^7^ revealed that 88% and 85% of the study's subjects would pay more money for their daughter and son, respectively, to have the laparoscopic pyloromyotomy because they prefer its cosmetic outcome over the open RUQ pyloromyotomy. This percentage would most likely increase if the parents were made aware that the initial incision from an open pyloromyotomy may grow significantly in length as the child ages, as evidenced by our case report.

## CONCLUSION

Significant scar growth from a standard RUQ pyloromyotomy incision was observed in the 2 patients who reached normal stature by adulthood, supporting the notion that scars resulting from surgical procedures in infancy grow more substantially with age than previously realized. This case series is small but has a perfect internal control of all being from the same family treated by the same surgeon, with those scars increasing in size until reaching adult stature. Although this case report did not assess the scar growth following an umbilical 5 mm port site from a laparoscopic incision, we believe that our results further support the public's preferential satisfaction with laparoscopy, given the dramatically smaller incisions at the time of surgery and the fact that scars can proportionally increase in size over time. It is critical to inform the patient's parents that the initial incision size will most likely not be the length of the final scar.

## Figures and Tables

**Table 1:** Family history*

Family member	Type of pyloromyotomy	Age when procedure was performed	Current age	Incision length at the time of procedure	Current length of scar
Grandmother	Oblique RUQ	4 wk	58 y	4 cm	12 cm
Aunt 1	Transverse RUQ	4 wk	23 y	4 cm	8 cm
Aunt 2	Transverse RUQ	6 wk	23 y	4 cm	4 cm
Patient	Laparoscopic converted to supraumbilical	4 wk	12 wk	3 cm	3 cm

*RUQ indicates right upper quadrant.
